# A Case of Epilepsy Apparently Due to Genital Irritation, and Cured by Circumcision

**Published:** 1887-04

**Authors:** Wharton Sinkler


### A CASE OF EPILEPSY APPARENTLY DUE TO GENITAL IRRITATION, AND CURED BY CIRCUMCISION.

By WHARTON SINKLER, M. D.

[Read before the Philadelphia Neurological Society.]

More or less difference of opinion exists as to the influence of peripheral irritation in the production of epilepsy. It is not generally agreed that sexual excesses or irritation of the generative apparatus can alone cause the epilepsy; but most writers admit that self-abuse or excessive venery bear a large part in the etiology of the disease, and that, if once established, these habits tend to aggravate and render it more difficult to cure.

Gowers thinks that masturbation in boys is a frequent cause of fits, which, while not genuine epileptic seizures, are convulsions of an hystero-epileptic kind.

Irritation of the prepuce or glans penis, even when not associated with masturbation, is believed by many to be the source of divers reflex nervous disorders, including epileptic convulsions, and many operations of circumcision have been done to relieve these conditions, with varying success.

To add some testimony to the subject, I wish to report to the Society the following case of marked epilectic fits associated with irritability of the genitals, and in which the attacks ceased after the operation of circumcision:

Frank P——, colored, æt. three years and five months, came to my clinic at the Infirmary for Nervous Diseases, July 19th, 1886.

His parents are healthy, and have five other children ; none of these have had fits, but one has had chorea. He was well until December, 1884, when he had two spasms. These were attributed to teething and taking cold. In January, 1885, he had another fit. The attack was marked, and lasted three or four minutes. Since that time he has averaged one fit a week. The attacks are severe, the convulsive movements being general, and afterwards he is dull and sleepy. For several days past he has had an attack every day.

The child is well nourished, and shows no sign of rickets except that he is backward in teething. He is intelligent and talks freely. The prepuce is elongated and inflamed at the edges, but can be retracted over the glans. Any touch, however, brings on an erection of the penis. The mother says that he handles the penis frequently, and seems to have some discomfort about it.

He was ordered potass. bromid. gr. 2 ter die. In one week returned and reported having had one attack. The bromide was increased to gr. 5 ter die.

August 2.—The boy has had thirty-five or forty attacks during the past week ; some days twelve or fifteen fits. He has become unable to speak, and seems to have lost his intelligence. The genitals are very irritable; a touch of the penis makes it rigid. Circumcision was advised, and the operation was performed by Dr. N. H. Goodman, August 9. The bromide was continued in 5-grain doses three times a day.

In the week after the operation he had two attacks. Since then there have been none. The bromide was taken for about three weeks.

I examined the child on December 27, and he seemed well. He was bright and active, and talked freely.

Gowers speaks of a case of epilepsy in a boy of thirteen years who had twelve or fourteen attacks a day under various treatment. It was found that he was addicted to self-abuse, and a blister on the prepuce reduced the fits to from two to seven a day. Circumcision was then performed, and the attacks ceased at once and did not recur. This author advises circumcision in all cases of epilepsy when masturbation is suspected.—*Polyclinic*.

